# Hypokalemic Hypophosphatemic Thyrotoxic Periodic Paralysis Associated with Bipolar Disorder Therapy

**DOI:** 10.7759/cureus.40988

**Published:** 2023-06-26

**Authors:** Talia E Rave, Marina Movshovich

**Affiliations:** 1 Department of Internal Medicine, New York Presbyterian Brooklyn Methodist Hospital, Brooklyn, USA; 2 Department of Endocrinology, New York Presbyterian Booklyn Methodist Hospital, Brooklyn, USA

**Keywords:** thyrotoxic periodic paralysis, hypokalemia, liothyronine, bipolar disorder, hyperthyroidism

## Abstract

Hypokalemic thyrotoxic periodic paralysis (TPP) is a rare complication of hyperthyroidism. TPP occurs due to the intracellular shift of potassium in the setting of elevated thyroid hormone. As potassium begins to be replenished, there is a risk of inducing hyperkalemia due to the extracellular shift of potassium. Therefore, it is recommended to replete potassium conservatively. There have been a number of studies reviewing the possible benefits of elevated thyroid hormone in treating bipolar disorder.

In this case report, a 37-year-old man with a past medical history of hypothyroidism and bipolar disorder presented with bilateral lower extremity paralysis. Liothyronine was added to his stable hypothyroid regimen for bipolar management. His initial labs on presentation were notable for severe hypokalemia, hypophosphatemia, and an undetectable thyroid-stimulating hormone (TSH). He was diagnosed with TPP, and his electrolytes were corrected with minimal repletion within 24 hours. More research is still required before concluding the role of thyroid hormone in mood disorders. This case report demonstrates a serious complication of supplemental thyroid hormone use. It is crucial to monitor thyroid function tests closely in order to avoid iatrogenic hyperthyroidism.

## Introduction

Thyrotoxic periodic paralysis (TPP) is a rare and life-threatening complication of hyperthyroidism. This condition is described as sudden onset weakness or paralysis, usually in the bilateral lower extremities, associated with hypokalemia. This is similar in presentation to familial hereditary periodic paralysis, which is a condition presenting with sudden paralysis associated with hypokalemia but is not associated with thyroid dysfunction [1]. TPP is resolved with the repletion of potassium. For patients at high risk of recurrent episodes, beta blockers such as propranolol can be used for prevention [2]. Thyroid hormone augmentation has been used to treat depression, and a number of studies have analyzed its potential benefits in treating rapid-cycling bipolar disorder [3]. We present the case of a young man with sudden bilateral lower extremity weakness and hypokalemia after liothyronine was added to his previously controlled hypothyroid disease regimen. His diagnosis of TPP exemplifies the potential side effects of iatrogenic hyperthyroidism.

## Case presentation

A 37-year-old Caucasian man with a past medical history of hypothyroidism and bipolar disorder presented to the emergency department (ED) with bilateral lower extremity weakness and tremors that had worsened over the past four hours. He had been stable on Armour thyroid (a combination medication of triiodothyronine (T3) and thyroxine (T4)) at 30 mg daily for the last few years, and his thyroid stimulating hormone (TSH) level four months prior was 1.9 uIU/mL. Three months prior, his psychiatrist added liothyronine (50 mcg daily) as an adjunctive medication for his bipolar disorder therapy. At that time, he was compliant with his prescribed medications, including lamotrigine 200 mg daily, divalproex 750 mg nightly, and Armour thyroid 30 mg daily. One month after starting liothyronine, the dose was increased to 125 mcg daily, and two months later, it was further increased to 150 mcg daily by his psychiatrist for his bipolar disorder treatment. Soon after the increased dose adjustment, the patient reported that he began to feel bilateral lower extremity weakness and tremors. Therefore, two days prior to presenting to the ED, the patient decreased the liothyronine to 75 mcg daily without consulting with his physician. On the day prior to his arrival at the ED, his lower extremity weakness and tremors significantly worsened. The patient denied any known allergies, family history, or surgical history. He denied tobacco use, illicit drug use, and alcohol use.

On presentation, he was found to have severe hypokalemia of 2.2 mmol/L and severe hypophosphatemia of 1.0 mg/dL. His TSH was undetectable at <0.005 uIU/mL. An electrocardiogram (ECG) was obtained, which was notable for an incomplete right bundle branch block, sinus tachycardia with a heart rate of 93 beats per minute, and an elevated QTc of 507. A head CT was obtained, which was notable for having no acute disease. The endocrinologist was consulted, and he was admitted to the telemetry unit for further cardiac monitoring considering his significant electrolyte abnormalities. Free triiodothyronine (T3) and free thyroxine levels were 1.9 pg/mL and 0.5 ng/dL, respectively. All labs obtained on presentation to the ED are represented below in Table 1.

**Table 1:** Lab results obtained on presentation to the emergency department

Test	Result	Reference range
Sodium	143 mmol/L	136 – 145 mmol/L
Potassium	2.2 mmol/L	3.5 – 5.1 mmol/L
Chloride	109 mmol/L	98 – 107 mmol/L
Carbon dioxide	23 mmol/L	21 – 32 mmol/L
Blood urine nitrogen	10 mg/dL	7 – 18 mg/dL
Creatinine	0.68 mg/dL	0.67 – 1.17 mg/dL
Glucose level	240 mg/dL	74 – 106 mg/dL
Anion gap	11 mmol/L	5 – 15 mmol/L
Calcium level	9.6 mg/dL	8.5 – 10.1 mg/dL
Phosphate	1.0 mg/dL	2.5 – 4.9 mg/dL
Magnesium	2.0 mg/dL	1.8 – 2.4 mg/dL
Total protein	7.0 g/dL	6.4 – 8.2 g/dL
Albumin	3.7 g/dL	3.4 – 5.0 g/dL
Total bilirubin	0.4 mg/dL	0.3 – 1.0 gm/dL
Bilirubin direct	0.1 mg/dL	0.0 – 0.3 mg/dL
Aspartate aminotransferase	21 unit/L	15 – 37 unit/L
Alanine aminotransferase	52 unit/L	8 – 62 unit/L
Alkaline phosphatase	103 unit/L	45 – 117 unit/L
White blood cells	6.04 x 10^3^/uL	3.98 – 10.04 x 10^3^/uL
Red blood cells	5.26 x 10(6)	4.63 – 6.08 x 10(6)
Hemoglobin	15.2 g/dL	13.7 – 17.5 g/dL
Hematocrit	42.4%	40.1 – 51.0%
Mean cell volume	80.6 fL	79.0 – 94.8 fL
Mean cell hemoglobin	28.9 pg	25.6 – 32.3 pg
Red cell distribution	11.4%	11.6 – 14.4%
Platelet	264 x 10^3^/uL	163 – 369 x 10^3^/uL
Mean platelet volume	10.1 fL	9.4 – 12.4 fL
Thyroid-stimulating hormone	<0.005 uIU/mL	0.360 – 3.740 uIU/mL
Free T3	1.9 pg/mL	2.5 – 4.3 pg/mL
Free thyroxine	0.5 ng/dL	0.9 – 1.7 ng/dL

Liothyronine and Armour thyroid were both held on admission. The patient received a total of 80 meq of potassium chloride and 15 mmol of potassium phosphorus within the first 24 hours of his admission. There was an improvement in his potassium and phosphorus levels to 4.6 mmol/L and 3.8 mg/dL, respectively.

Within three days, the patient continued to have mild weakness in his bilateral lower extremities; however, he had significant improvement in muscle strength and was able to ambulate. His weight-based dose of levothyroxine was calculated to be 125 mcg-150 mcg. However, due to his current thyroid labs, he was prescribed levothyroxine 25 mcg and scheduled for a close follow-up with the endocrinologist after discharge.

The patient had an outpatient visit with the consulted endocrinologist a week and a half after discharge. He had no complaints, and he reported that his bilateral lower extremity weakness had fully resolved. He denied constipation, diarrhea, heat or cold intolerance, weight loss, weight gain, or palpitations. Repeat TSH was mildly elevated to 3.87 uIU/mL, free thyroxine was mildly depressed to 0.8 ng/dL, and free T3 was within normal limits at 2.6 pg/mL. The patient was advised to take 25 mcg of levothyroxine Monday through Friday and 50 mcg Saturday and Sunday.

The patient returned for a follow-up visit six weeks later. Repeat serum lab results were notable for TSH at 2.5 uIU/mL, free thyroxine at 1.4 ng/dL, free T3 at 3.7 pg/mL, and thyroid peroxidase antibody at 1.0 IU/mL. The patient endorsed feeling well with no complaints about his current levothyroxine regimen.

## Discussion

The hypokalemia seen in thyrotoxic periodic paralysis (TPP) is due to an intracellular shift of potassium rather than urinary or fecal potassium loss. Therefore, in most cases, the potassium levels normalize quickly. One retrospective case review of 24 episodes of TPP in 19 patients found an average of 1.5-10 hours until potassium levels normalized, with an average of 40-200 mmol of potassium chloride required for treatment [4]. It is important to note that more than 40% of patients in this case review had rebound hyperkalemia [4]. A suggested protocol is to treat the hypokalemia seen in TPP conservatively with 30 mEq of oral potassium every two hours and not exceed 90 mEq in 24 hours [5]. As expected, our patient had a quick resolution of his potassium. Serum phosphate levels are also often depleted in patients with TPP. In the case review by Manoukian et al., 80% of cases reviewed presented with hypophosphatemia, with only 33% requiring replacement therapy [4]. Many of these cases showed a significant elevation in phosphorus levels after the resolution of paralysis, even in those who did not receive repletion [4]. Similarly, our patient had a phosphate level of 1.0 mg/dL that increased to 3.8 mg/dL after only 15 mmol of potassium phosphate.

The intracellular shift of potassium seen in TPP occurs due to the increase in beta-adrenergic stimulation from elevated circulating thyroid hormone. This causes activation of Na/K-ATPase channels in skeletal muscle membranes, causing a large intracellular potassium shift leading to hypokalemia, hyperpolarization, and eventually periodic paralysis [1,6]. This phenomenon can be precipitated by a high-carbohydrate diet, which increases adrenergic activity and insulin release, ultimately leading to Na/K-ATPase activity [6]. Another player in the increased activity of the Na/K-ATPase channels is thyroid hormone-responsive elements (TREs), which are involved in transcribing a subunit of the Na/K-ATPase channel and are stimulated by elevated levels of thyroid hormone [6]. Although hyperthyroidism has a higher incidence in women, TPP is more frequently seen in men. This disparity could be due to the stimulation effect testosterone has and the inhibition effect estrogen and progesterone have on the activity of Na/K-ATPase channels [6-8].

Multiple ECG changes can be seen in hypokalemic periodic paralysis that range from non-threatening to life-threatening in severity. These include Supraventricular arrhythmias, first- and second-degree heart block, right bundle branch block, QRS and QT interval prolongation, and T wave flattening and inversion [9]. Although rare, ventricular tachycardia and cardiac arrest have also been documented in TPP cases [9]. Our patient had an incomplete right bundle branch block and an elevated QTc. Once his potassium level was normalized, his ECG returned to his baseline normal sinus rhythm.

It is important to note that prior to our patient's episode of TPP, he was prescribed liothyronine, in addition to his Armour thyroid regimen, for potential improvement in his bipolar disorder by his outpatient psychiatrist. There are multiple reports highlighting the benefit of supraphysiologic levothyroxine levels as adjunctive therapy for treatment-resistant depressive symptoms of bipolar disorder [10]. Sienaert et al. did a literature review on the use of T3 in the treatment of bipolar depression and found that although there were few available studies, many of which were small and flawed, there were clues suggesting that T3 could augment antidepressants and lithium and possibly protect against rapid cycling bipolar disorder [11]. Seshadri et al. performed a systemic review, finding a significant improvement in bipolar disorder symptoms in open-label trials; however, these results were not replicated in randomized controlled trials [3].

## Conclusions

As physicians, we are obligated to treat our patients while also balancing our oath to do no harm. In this case, there were two instances where this balance was challenged. The first instance highlights the importance of conservative potassium repletion in TPP. It is crucial to diagnose TPP early. The etiology of severe hypokalemia in these cases is due to the intracellular shift of potassium. Therefore, repleting potassium too aggressively can cause rebound hyperkalemia, which is a serious and possibly fatal risk. The second instance emphasizes the significance of medication side effects. In this case, our patient presented to the ED with TPP provoked by iatrogenic hyperthyroidism. More research is necessary before concluding that supratherapeutic thyroid hormone has a significant role in mood disorder management. If thyroid hormone will be used for mood disorder therapy, especially in patients with underlying thyroid disease, it is imperative to frequently monitor thyroid function tests in order to prevent the serious complications of hyperthyroidism.
